# Characterization of regulatory T cells in SARS-CoV-2 infected hemodialysis patients: relation to clinical and radiological severity

**DOI:** 10.1186/s12882-022-03024-x

**Published:** 2022-12-07

**Authors:** Emad Samaan, Marwa O Elmaria, Doaa Khedr, Tamer Gaber, Ahmed G Elsayed, Ragy N Shenouda, Hend Gamal, Doaa Shahin, Nashwa K Abousamra, Rasha Shemies

**Affiliations:** 1 Mansoura Nephrology and Dialysis Unit, Internal Medicine Department, Faculty of Medicine, Mansoura University, El Gomhoria St, Mansoura, 35516 Egypt; 2 Chest Department, Faculty of Medicine, Mansoura University, Mansoura, Egypt; 3 Diagnostic and Interventional Radiology Department, Faculty of Medicine, Mansoura University, Mansoura, Egypt; 4 Medical Microbiology and Immunology Department, Faculty of Medicine, Mansoura University, Mansoura, Egypt; 5 Haematology Unit, Clinical Pathology Department, Faculty of Medicine, Mansoura University, Mansoura, Egypt

**Keywords:** CD39+ Tregs- COVID 19- Hemodialysis- T regulatory cells- SARS-CoV-2

## Abstract

**Background:**

Disordered Treg counts and function have been observed in patients with SARS-Cov-2 and are thought to contribute to disease severity. In hemodialysis patients, scarce data are available on the Treg response to SARS-CoV-2 or its relation to the clinical presentation.

**Methods:**

A cross-sectional study included one hundred patients divided into three groups, thirty SARS-CoV-2-infected hemodialysis patients (COV-HD), and thirty confirmed SARSCoV-2 infected patients (COV), and forty non-infected hemodialysis patients (HD). Flow cytometric analysis of CD4, CD25, FoxP3, and CD39+ Tregs was done for all patients and tested for correlation to in-hospital mortality, clinical, radiological severity indices.

**Results:**

COV-HD and COV patients had significantly lower Treg cell count than HD patients (Median value of 0.016 cell/ μl vs 0.28 cell/ μl, respectively- P: 0.001). COV-HD patients had higher CD39+ Tregs (median value of 0.006 cell/ μl vs 0.002 cell/ μl, respectively- P: 0.04). COV-HD patients had significantly lower hospital stay (median value of 3 vs 13 days, P:0.001), ICU admission rates (26.5% vs 46.7%, P:0.005) and in-hospital mortality (20.7% versus 43.3%, P:0.003) than COV patients. Treg and CD39 expressing Treg counts were not correlated to severity indices in both groups. A high neutrophil to lymphocyte ratio is strongly correlated to disease severity in COV-HD patients.

**Conclusions:**

This study provides evidence of T-cell, particularly T-regulatory cell decline in SARS-CoV-2 and suggests that hemodialysis per se does not distinctively impact the T-cell response. COV-HD patients exhibited a higher CD39+ Treg count and a better clinical profile, however, larger studies are needed to extrapolate on these findings.

## Background

The host response to viral infection, particularly the cellular immune response is mandatory to control viral replication and enhance viral clearance. In patients with Severe acute respiratory syndrome coronavirus 2 (SARS-CoV-2) infection, low levels of T-cells have been reported in several studies, this lymphopenia is thought to result from the recruitment of T cells to the site of infection [1]. As a subset of CD4+ T cells, T regulatory cells (Tregs) are responsible for counteracting the overactivation of the immune response which is a hallmark of SARS-CoV-2 [2]. Altered absolute and relative numbers of Treg counts have been observed in patients with severe SARS-CoV-2 and are thought to contribute to the pathogenesis of immune hyperreactivity [3]. This controversy as regard the number and function of Tregs in SARS-CoV-2 patients is due to the the different identification methods of Tregs which vary between the phenotypic and functional assessment [4]. Scarce data on the immune response to SARS-CoV-2 in hemodialysis patients are currently available. Basically, the accumulation of uremic toxins was reported to be associated with impaired or dysregulated immune response, both innate and adaptive immunity [5, 6]. A lower thymic output and premature aging of the T-lymphocytes have been already reported [5–7], which in turn increases the susceptibility to infections including pulmonary infections. Earlier studies have reported a 15-fold increase in mortality in hemodialysis patients due to respiratory tract infections [8]. In the era of SARS-CoV-2, hemodialysis patients exhibit greater risks for SARS-CoV-2 infection as well as increasing disease severity. The mortality rates of infected HD patients were expected also to be high. Conversely, a study from china reported that HD patients had comparable mortality and fewer ICU admissions when hospitalized with SARS-CoV-2 [9]. This may be related to the extracorporeal therapies that have been studied as potential treatments for the removal of cytokines in critically ill patients with SARS-CoV-2 [10, 11]. Also, it was hypothesized that constant repeated antigenic stimulation of T lymphocytes in patients undergoing hemodialysis may accelerate the aging of the immune system, all of which could affect the immune response and hence the clinical outcomes in hemodialysis patients with SARS-CoV-2 [12]. Tregs, the key mediators of immune homeostasis, are of great functionality in end stage kidney disease (ESKD) patients and be remarkably decreased in hemodialysis patients before the pandemic of SARS-CoV-2 [5]. Notably, Tregs served as a protective measure against progressive renal fibrosis in chronic kidney disease by suppressing the production of proinflammatory cytokines and decreasing the effector phenotype [8, 10–12]. Tregs express CD25 and FOXP3 which is a transcriptional regulator that controls the development and function of Tregs and is expressed by almost all inhibitory Tregs [13]. CD39 is predominantly expressed on human CD4+ Foxp3+ T cells. It has revealed a number of neoteric functions in close relation with Tregs and may be an indispensable chip to identify Tregs [14]. CD39 is considered a functional Treg marker as it directly contributes to the suppressive capacity of Tregs. Some studies have reported that CD39+ Tregs have more immunosuppressive effects than CD39- Tregs [15]. Currently, the influence of renal insufficiency and subsequent hemodialysis on the expression and differentiation of Tregs in SARS-CoV-2 infected hemodialysis patients is a non-settled issue. This study aims to evaluate the T lymphocytes’ expression patterns especially Tregs and the level of CD39 expressing Tregs in SARS-CoV-2 infected hemodialysis patients. Also, testing their relation to in-hospital mortality, clinical and radiological severity indices.

## Materials and methods

This is a cross-sectional study carried out at Mansoura University Hospitals, Mansoura University, during the period between April and December 2021.

### Patient selection

The study population included one hundred patients divided into three groups for comparison. Thirty hemodialysis patients with confirmed SARS-CoV-2 (COV-HD group), thirty confirmed SARS-CoV-2 infected patients (COV Group), and 40 non-infected hemodialysis patients (HD Group). The three groups were compared regarding clinical, laboratory, radiological, and outcome data. All patients were consented for participation in the study.

### Sample size calculation

Sample size was calculated by PASS software for Windows, version 11.0.8. PASS 11. NCSS, LLC. Kaysville, Utah, USA. (www.ncss.com). Calculation relied upon a previous study that characterized Tregs in HD patients [16]. Group sample sizes of 30 and 30 achieve 85% power to reject the null hypothesis of zero effect size when the population effect size is 0.8 and the significance level (alpha) is 0.050 using a two-sided two-sample equal-variance t-test. Another control group of non-infected HD patients was included for comparison.

### Inclusion and exclusion criteria

SARS-COV-2 infected patients were included based on a confirmed real-time reverse transcription-polymerase chain reaction (rRT-PCR) test for the qualitative detection of the nucleic acid in a nasopharyngeal swab. The exclusion criteria were restricted to the following: Patients who refused the enrolment in the study, patients with a history of autoimmune disease, active malignancy, and patients on current immune-modulatory drugs.

### Data collection

Data of COV-HD and COV patients were collected from cases admitted to Mansoura University Hospital- Isolation Section. Data from HD patients were collected from Mansoura Nephrology and Dialysis, Mansoura University, Egypt. The time of data collection was 8 months.

Demographic and clinical data: Epidemiological characteristics, comorbidities, coagulation function markers on admission, and disease severity, were obtained from patients’ medical records. The SARS-CoV-2 severity was judged according to the need for hospitalization, duration of hospital stays, oxygen saturation at time of admission, need for oxygen supplementation, and in-hospital mortality.

### Blood sampling and laboratory

For HD patients, blood samples were collected from the arteriovenous fistula just before starting the HD session. Routine laboratory tests were performed within the days of blood sampling using an automated analyser, complete blood picture, differential leucocytic count, and D-dimer test.

#### Flow cytometric T-reg cell analysis

Flow cytometry was performed on a FACSCanto II flow cytometer (BD Biosciences, USA).

**Fig. 1:**
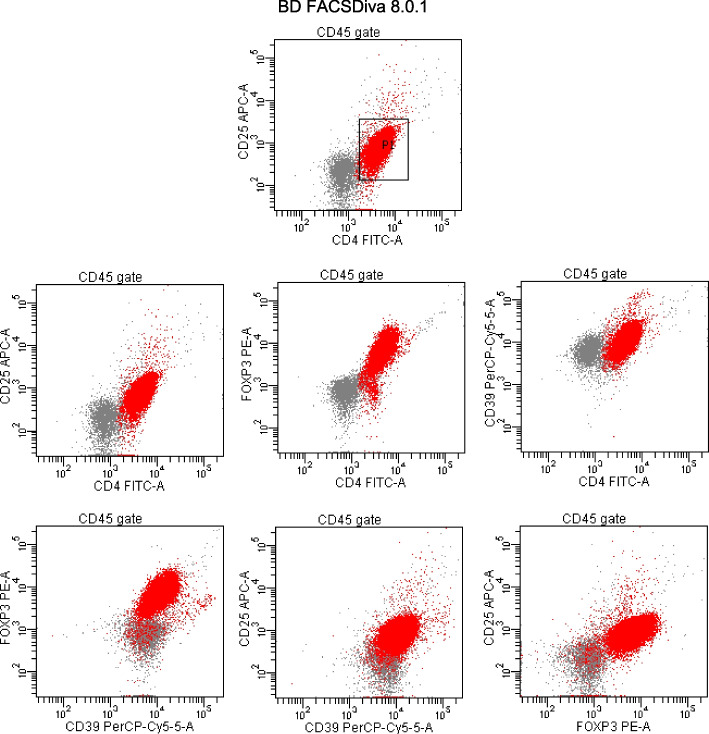
A representative flowcytometric plot demonstrating FoxP3 versus CD25 and CD39 expression on CD4+ T cells

Briefly, 50 μl of fresh whole blood was incubated with the appropriate amounts of fluorochrome-labelled monoclonal antibodies CD45 Krome Orange (clone J33), CD4 FITC (clone 13B8.2), CD25 APC (clone IHT44H3) and CD39 PC5.5 (clone BA54) from Beckman Coulter, France. Incubation was done at room temperature in the dark for 15 min using appropriate mouse immunoglobulin isotypes as a control. Following incubation, 1 ml erythrocyte lysing solution (VersaLyse, Beckman Coulter) was added to the samples and incubated under the same conditions for 20 min. Then, cells were fixed and permeabilized (using intraprep permeabilization reagent, Beckman Coulter), followed by intracellular staining with anti-FoxP3-PE (clone 259D, Beckman Coulter, France) for 30 min. Cells were resuspended in PBS and analysed. Finally, the cells were characterized by flow cytometry analysis using BD FACEDiva Software. Analysis performed with CD4 + CD25 + CD39+ FOXP3+ T-reg cells expressed as a percentage of the whole CD4 subset (Fig. 1).

### Chest radiography

A Spiral CT scan was done for all patients from the root of the neck to the level of the upper pole of the kidneys during a single-breath hold using a 1 mm slice thickness. Images were reconstructed in axial, coronal, and sagittal reformats with standard pulmonary filtering. Several studies have been published reporting chest CT findings in SARS-CoV-2. in our study, we used total severity score (TSS).

### Total severity score [17]

In this score, five lobes of the lungs were assessed for ground-glass opacities, mixed ground-glass opacities, or consolidation. Each lobe given 0 to 4 points, depending on the percentage of the involved lobe: 0 (0%), 1 (1–25%), 2 (26–50%), 3 (51–75%), or 4 (76–100%). The total severity score (TSS) is calculated by summing the points from each of the five lobes. The TSS cut-off for identifying the severe-critical type of 7.5.

### Quantitative analysis of ground glass percentage

All digital imaging and communications in medicine (DICOM) data of thin cut CT chest were analyzed using Synapse 3D Fujifilm Medical Systems version 3.5 on specific workstations for automated analysis calculate ground-glass opacities (GGOs).

The data sets include 4 groups of density ranges: red color representing emphysema: From − 1024 to − 950 HU, Yellow color representing normal lung: From − 949 to − 750 HU, Blue color representing GGOs: From − 749 to − 300 HU, and violet color representing consolidation: From − 299 to + 40 HU.

Additionally, CT was assessed for the presence of other CT findings associated with COVID-19 such as consolidations, crazy-paving, subpleural bands, vascular dilatation, and reverse halo signs as well as atypical features of COVID-19 such as pulmonary nodules, mediastinal lymphadenopathy, and pleural effusion with mention of laterality, lobar affection (upper or lower), and distribution pattern (peripheral, central, or diffuse).

**Fig. 2:**
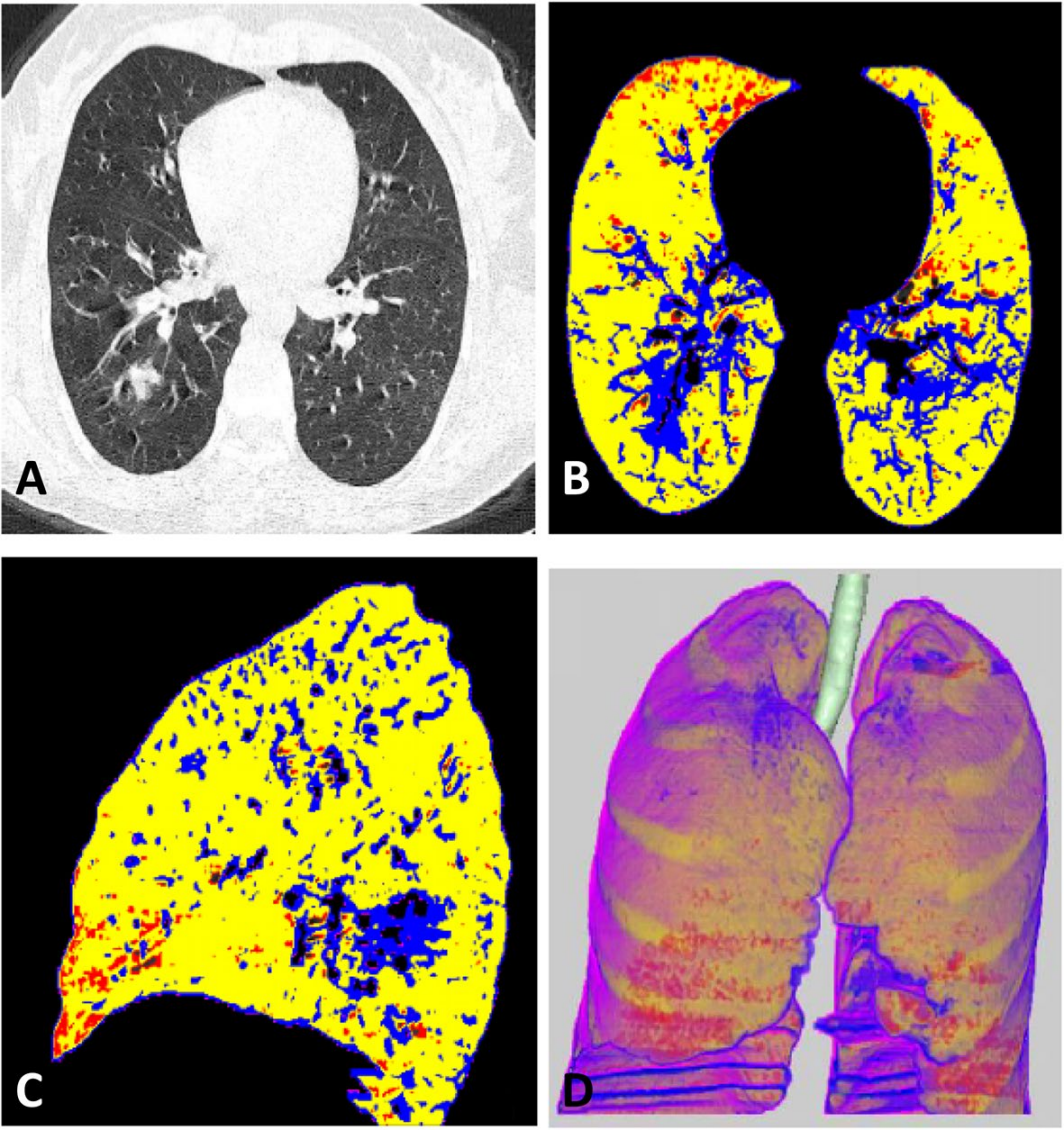
CT findings of female patient with mild disease, Total severity score (TSS) equal 4. **A** Axial CT image shows right lower lobe ground glass density. **B** and **C** Axial and sagittal colored analysis of the lung density revealed mild ground glass percentage (20%) in blue color compared to normal lung density which is represented with yellow color. **D** 3D image analysis of the lung with different densities

**Fig. 3:**
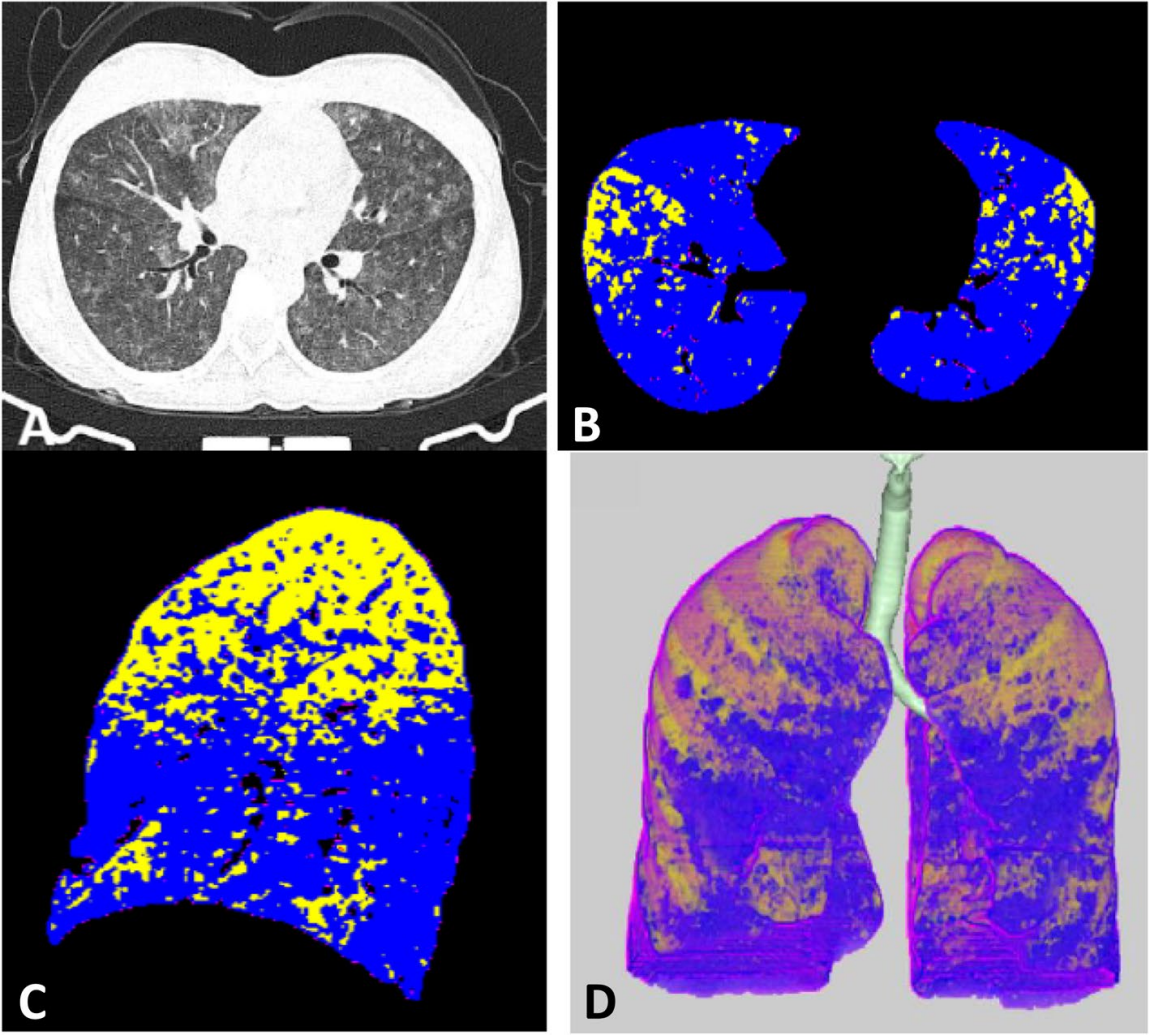
CT findings of Male patient with marked disease, Total severity score (TSS) equal 18. **A** Axial CT image shows multiple ground glass densities seen scattered in the scanned lungs. **B** and **C** Axial and sagittal colored analysis of the lung density revealed marked ground glass percentage (70%) in blue color compared to normal lung density which is represented with yellow color. **D** 3D image analysis of the lung with different densities

Figures 2 and 3 show the radiological differentiation of disease burden between mild and severe cases.

### Statistical analysis

The collected data were coded, processed & analysed using the Statistical Package for Social Science (SPSS) version 25 for Windows on personal computers. Qualitative information was described as percentages and numbers. While quantitative information was described as means [± standard deviation (SD)] for normally distributed variables or medians (interquartile range; minimum-maximum), for non-normally distributed data. To assess the normality of the distribution of variables, the Shapiro-Wilk test was used. For comparing the groups, the t-test was used for normally distributed data of the variables of two groups, while the Mann-Whitney test was used for non-normally distributed data of variables of two groups. A Chi-square test was done for comparing qualitative variables’ data between the study groups. The correlation statistics were done by Spearman’s rank test. The level of significance will be considered at 5% (*P* ≤ 0.05).

## Results

### SARS-CoV-2 infected patients (COV-HD+ COV groups) in comparison to the HD group

SARS-CoV-2 infected patients had significantly lower WBCs total and differential counts than the HD group (Table 1). CD4+ T cells and T regulatory cells were also significantly lower in all COV cases whether they are on hemodialysis or not than non-infected HD group [45.1 cell/ μl (1.9–397.6) versus 125.2 cell/ μl (7.3–1162.9), P 0.001, and 0.016 cell/ μl (0–5.77) versus 0.28 cell/ μl (0–140.9), *P* < 0.001, respectively]. Neutrophil to lymphocyte ratio was significantly higher in SARS-Cov2 patients [3.44 (1.44–21.7) versus 1.88 (0.7–4.99) in HD patients, *P* < 0.001] (Table 1).

**Table 1:** Comparing demographic and laboratory data between all Cases of COVID (COV-HD+ COV groups) and control (HD group)

	Case (COV-HD+ COV groups)	Control (HD group)	***P*** Value
Number	60	40	
Gender			
Male	30 (50)	17 (42.5%)	0.54 ^a^
Female	30 (50)	23 (57.5%)	
**CBC differential counts**			
Hemoglobin (mg/dl)	10.3 (6.5–17.6)	10.1 (7.7–14.8)	0.28 ^a^
Total WBCs (cell/ μl)	7600 (3200–22,500)	6350 (2300–10,200)	**0.02** ^a^
Neutrophil (cell/ μl)	5402 (2080–18,225)	3270 (782–6796)	**< 0.001** ^a^
Lymphocyte (cell/ μl)	1137 (480–5000)	1722 (665–3276)	**0.002** ^a^
NL ratio (%)	3.44 (1.44–21.7)	1.88 (0.7–4.99)	**< 0.001** ^a^
Flowcytometry counts			
CD4+ T cells (cell/ μl)	45.1 (1.9–397.6)	125.2 (7.36–1162.9)	**0.001** ^a^
Tregs (cell/ μl)	0.016 (0–5.77)	0.28 (0–140.9)	**< 0.001** ^a^
CD39+ Tregs (cell/ μl)	0.0031 (0–5.6)	0.049 (0–135.3)	**0.001** ^a^

Non-parametric data: Median (25–75%)

Nominal data: Number (Percentage)

^a^Mann-Whitney U test

### SARS-Cov2 infected hemodialysis (COV-HD group) in comparison to infected patients, not on regular hemodialysis (COV group)

COV-HD and COV groups were comparable in their WBCs total and differential counts, CD4+ T cells, and T regulatory cells, however, there was a significantly higher CD39 expression on T regulatory cells in COV-HD patients than in COV patients [0.006 cell/ μl (0–5.6) versus 0.002 (0–0.14), P 0.04]. COV-HD patients had significantly shorter hospital stay [3 (0–12) days versus 15 (3–33) days, P 0.001], better oxygen saturation at admission [89% (72–96%) versus 88% (60–99%)], less ICU admission (26.5% versus 46.7%, P 0.009) and in-hospital mortality rates (23.5% versus 43.3%, P 0.005) than COV group. D-dimer levels were significantly higher in COV-HD than in COV patients (Table 2).

**Table 2:** Comparing demographic and laboratory data between COV and COV-HD patients

	COV group (***n*** = 30)	COV-HD group (***n*** = 30)	***P*** Value
Demographic and Clinical data			
**Age**	60.6 (13.6)	54 (8.9)	0.115 ^a^
**Gender**			
**Male**	9 (30)	21 (70)	**0.006**
**Female**	21 (70)	9 (30)	
**Diabetes**	14 (46.7)	13 (43)	0.5
**Number of days of hospital admission**	15 (3–33)	3 (0–12)	**0.001** ^a^
**O2 Saturation at admission**	88 (60–99)	89 (72–96)	**0.008** ^a^
**Place of stay (OPC, Ward, ICU)**			
**OPC**	2 (6.7)	12 (40)	**0.009**^**c**^
**Ward**	14 (46.7)	10 (33.5)	
**ICU**	14 (46.7)	8 (26.5)	
**Outcome**			
**OPC**	1 (3.3)	11 (36.5)	**0.005**^**c**^
**Discharge**	16 (53.3)	12 (40)	
**Death**	13 (43.3)	7 (23.5%)	
Radiological Data			
**GGO percentage%**	50 (0–86)	45 (0–69)	0.19 ^a^
**Total severity score in CT (TSS) (out of 20)**	7.5 (2–15)	7 (2–15)	0.12 ^a^
**TSS (Severe > 7.5)**	16 (53%)	10 (33%)	0.08 ^a^
Laboratory data			
CBC and Coagulation			
**Hemoglobin (mg/dl)**	11.25 (6.5–17.6)	9.5 (7.8–12.7)	**< 0.001** ^**a**^
**Total WBCs (cell/ μl)**	8616 (3200–20,800)	7300 (3560–22,500)	0.423^a^
**Neutrophil (cell/ μl)**	6790 (2080–14,890)	4346 (2242–18,225)	0.152 ^a^
**Lymphocyte (cell/ μl)**	1100 (500–3600)	1204 (480–5000)	0.9 ^a^
**NL ratio**	5.6 (1.81–13.22)	2.8 (1.84–21.75)	0.132 ^a^
**D-dimer (ng/ml)**			
**Less than 200**	19 (63.3%)	13 (43.3)	**0.04** ^a^
**200–500**	8 (26.7%)	4 (13.3)	
**More than 500**	3 (10%)	13 (43.3)	
Flowcytometry Data			
**CD4+ T cells (cell/ μl)**	33.8 (2.2–359.1)	52.16 (1.92–399.6)	0.4 ^a^
**Tregs (cell/ μl)**	0.011 (0–0.33)	0.028 (0–5.77)	0.1 ^a^
**CD39 expressing Tregs (cell/ μl)**	0.002 (0–0.14)	0.006 (0–5.6)	**0.04** ^**a**^

Non-parametric data: Median (25–75%)

Nominal data: Number (Percentage)

^a^ Mann-Whitney U test

^C^ chi square test

### Correlation statistics of different T cell population counts with the clinical and radiological severity criteria

#### In COV-HD group

Lymphopenia is significantly correlated to degree of hypoxemia (R = 0.5, P 0.004), higher rates of ICU admission (R = − 0.36, P 0.04), radiological severity in CT chest score (R = − 0.41, P 0.02- R = − 0.45, P 0.01). Neutrophil to lymphocyte ratio (NLR) is significantly correlated positively to all radiological and clinical severity parameters including the in-hospital mortality (Table 3).

**Table 3:** Correlation statistics of different T cell population counts with the clinical and radiological severity criteria in COV-HD and COV patients

		Clinical Severity Parameters				Radiological Severity Parameters	
		Number of days of admission	Hypoxemia	ICU Admission	In-Hospital Mortality	TSS CT^a^	Percentage of GGO^a^
**COV-HD**	**Lymphocytes**	R = − 0.33^s^ P 0.07^s^	**R = − 0.5**^**s**^ **P 0.004**^**s**a^	**R = − 0.36**^**s**^ **P 0.04**^**s**a^	R = − 0.3^s^ P 0.09^s^	**R = − 0.41**^**s**^ **P 0.02**^**s**a^	**R = − 0.45**^**s**^ **P 0.01**^**s**a^
	**NLR**^a^	**R = 0.4**^**s**^ **P 0.02**^**s**a^	**R = 0.54**^**s**^ **P 0.002**^**s**a^	**R = 0.64**^**s**^ ***P*** **< 0.001**^**s**a^	**R = 0.71**^**s**^ ***P*** **< 0.001**^**s**a^	**R = 0.61**^**s**^ ***P*** **< 0.001**^**s**a^	**R = 0.63**^**s**^ ***P*** **< 0.001**^**s**^
	**CD4+ T cells**	R = − 0.14^s^ P 0.4^s^	R = 0.26^s^ P 0.1^s^	R = 0.18^s^ P 0.3^s^	R = − 0.1^s^ P 0.3^s^	R = − 0.25^s^ P 0.1^s^	R = − 0.2^s^ P 0.2^s^
	**Tregs**	R = 0.1^s^ P 0.4^s^	R = 0.1^s^ P 0.4^s^	R = 0.1^s^ P 0.4^s^	R = 0.1^s^ P 0.4^s^	R = 0.1^s^ P 0.3^s^	R = 0.2^s^ P 0.2^s^
	**CD39 Expressing Tregs**	R = 0.09^s^ P 0.6^s^	R = 0.05^s^ P 0.08^s^	R = 0.1^s^ P 0.5^s^	R = 0.1^s^ P 0.3^s^	R = 0.1^s^ P 0.5^s^	R = 0.1^s^ P 0.3^s^
**COV**	**Lymphocytes**	R = 0.04 ^s^ P 0.85 ^s^	R = − 0.06 ^s^ P 0.7 ^s^	R = 0.02 ^s^ P 0.8 ^s^	R = 0.004 ^s^ P 0.9 ^s^	R = 0.18^s^ P 0.3^s^	R = 0.31^s^ P 0.08^s^
	**NLR**	R = 0.1 ^s^ P 0.5 ^s^	R = 0.06 ^s^ P 0.7 ^s^	R = 0.29 ^s^ P 0.1 ^s^	R = 0.13 ^s^ P 0.4 ^s^	R = 0.06 ^s^ P 0.7 ^s^	R = −.09 ^s^ P 0.6 ^s^
	**CD4+ T cells**	**R = − 0.4**^**s**^ **P 0.03**^**s**a^	R = − 0.1^s^ P 0.5^s^	R = − 0.2^s^ P 0.2^s^	**R = − 0.54**^**s**^ **P 0.003**^**s**a^	**R = − 0.53**^**s**^ **P 0.003**^**s**a^	R = − 0.01^s^ P 0.9^s^
	**Tregs**	R = 0.09^s^ P 0.6^s^	R = − 0.05^s^ P 0.7^s^	R = 0.1^s^ P 0.4^s^	R = − 0.3^s^ P 0.06^s^	R = − 0.2^s^ P 0.3^s^	R = − 0.06^s^ P 0.7^s^
	**CD39 Expressing Tregs**	R = −0.01^s^ P 0.9^s^	R = − 0.06^s^ P 0.7^s^	R = 0.3^s^ P 0.08^s^	R = − 0.1^s^ P 0.5^s^	R = − 0.14^s^ P 0.4^s^	R = − 0.07^s^ P 0.7^s^

R: correlation coefficient (^**S**^: spearman rank test)

*NLR* Neutrophil to lymphocytes ratio

*TSS CT* Total severity score in chest computed tomography

*GGO* Ground Glass Opacity

*P P* value

^a^significant correlations

#### In COV group

CD4+ T cell counts are correlated negatively to the length of hospital stay (R = − 0.4, P 0.03), total severity CT chest score (R = − 0.53, P 0.003), and in-hospital mortality (R = − 0.54, P 0.003). lymphopenia, T reg, and CD- 39 expressing Treg counts are not correlated to any parameter of the clinical and radiological severity (Table 3).

## Discussion

Patients on maintenance hemodialysis are particularly susceptible to SARS-CoV-2 infection due to uremia-related immune dysfunction, enhanced comorbidity burden, and the risk of cross-contamination in hemodialysis units. It is unknown whether hemodialysis patients represent a distinctive group of patients with a distinct immune response to SARS-CoV-2. It is recognized that the T- lymphocyte count is considerably decreased in patients with SARS-CoV-2 as well as in HD patients on an individual basis [18, 19]. This lymphopenia appears more manifest in patients with severe disease [18–20]. In a case series of five hemodialysis patients who contracted SARS-CoV-2, lymphopenia was reported in all cases with pulmonary ground-glass opacities as the most common radiologic findings [21]. Notably, earlier studies have found that decreased lymphocyte count is a clinical predictor of mortality due to SARS-CoV-2 infection [22, 23]. This observation was further confirmed in the study of Wang et al., who revealed that increased counts of circulating lymphocytes in elderly patients with SARS-CoV-2 were predictive of a better outcome [24]. Similarly, The NLR has been considered an independent biomarker for predicting poor clinical outcomes [25]. Comparably the present study reported significantly reduced lymphocyte count in COV-HD and COV patients. The NLR was significantly higher in COV-HD and COV patients compared to patients in the HD group. The NLR positively correlated to the TSS score and percent of ground-glass opacities while negatively correlated to the oxygen saturation, hospital stay, ICU need, and in-hospital mortality in COV-HD patients, this was not applied to COV patients. Tregs alterations have been largely observed in patients with SARS-CoV-2, recent studies have demonstrated a significant reduction in Treg numbers in SARS-CoV-2 patients, which has been associated with increasing disease severity and increased risk of respiratory failure [26, 27]. Meckiff et al. presented a single-cell transcriptomic analysis of more than 100,000 viral antigen-reactive CD4 + T cells from 40 patients with SARS-CoV-2 and found a decreased proportion of SARS-CoV-2-reactive regulatory T cells [26]. A notable reduction in the frequency of Tregs as well as the expression levels of correlated factors FoxP3, transforming growth factor-β [TGF-β], and IL-10) in the SARS-CoV-2 ICU patients has been reported [27]. On the other side, many studies confirmed increased Treg counts in mild, moderate [28], and severe [29] forms of SARS-CoV-2 infection. Another report recorded elevated levels of activated Tregs in early asymptomatic SARS-CoV-2 infection despite the normal total number of Tregs [30]. The present study showed a significantly reduced total number of CD4+ lymphocytes and Tregs in patients with SARS-CoV-2 (COV-HD + COV) than in non-infected HD patients suggesting that the observed alterations in CD4 lymphocyte and neutrophil counts resulted from a SARS-CoV-2 effect rather than an HD effect. Moreover, it is noteworthy to point out an unusual phenotype of Tregs which has been recently identified in patients with severe SARS-CoV-2 disease and is largely similar to that identified in tumor-infiltrating Tregs which expresses proinflammatory mediators and is mostly associated with poor outcomes [3, 31] but unfortunately, our study did not permit further investigation on this phenotype. CD 39 has an important role in the anti-inflammatory effect of Treg [15]. A previous study demonstrated that CD 39+ cell count was significantly higher in SARS-CoV-2 infected adult patients versus a healthy control group, an observation that is not applied to a juvenile cohort in the same study [32]. In our study, CD 39+ Tregs were significantly lower in SARS-CoV-2 infected groups than in the non-infected ones, although they were found to be significantly higher in COV-HD patients in a subgroup analysis. Being applied to the general population with healthy controls, it is difficult to compare our findings on HD patients with their results. Another area of confusion is the mortality risk in HD patients with SARS-CoV-2 infection. Some previously published reports showed high mortality rates among maintenance hemodialysis patients hospitalized for SARS-CoV-2 [33–36]. Some other studies demonstrated that chronic inflammation, a salient feature in HD patients, might protect those patients from severe COVID-19-related symptoms, ICU admission, and mortality [9, 16]. These discordant results are accepted because of the limitations known in the observational studies and multiple confounders which can affect the patient prognosis like age [37], associated comorbidities, and selection bias. The present study revealed a better survival rate, less severe hypoxemia indicating hospital or ICU admission in COV-HD than COV patients despite the comparable age, comorbidity, radiological severity criteria. This may be attributed to many factors: the fact that HD patients are more monitored compared to non-HD leading to early identification and management of infection, lessened severity indices of patients included in the COV-HD group, or perhaps the consequence of more frequent cytokine clearance in regular HD sessions. Whether or not CD 39+ Tregs contributed to a better clinical profile in our COV-HD patients, this still needs furher exploration on larger sized samples. A larger sample size is needed for performing multivariate analyses as well. We admit other three important limitations: (1) the study did not include a helathy control group from the general population for comparison; (2) the data of the drug therapy was not analyzed; (3) suppression of T cell proliferation is needed for a better evaluation of Treg.

## Conclusions

This study was carried out to evaluate the effect of hemodialysis on T-regulatory cells in SARS-COV-2 infected patients, and provide evidence of T-cell, particularly T-regulatory cell decline in hemodialysis patients with SARS-COV-2 and suggest that hemodialysis per se does not distinctively impact the T-cell response in patients with SARS-CoV-2. Therefore, the T-cell targeted therapies for SARS-CoV-2 in the general population may be effectively used in hemodialysis patients. The role of CD39 expressing Tregs and Treg phenotyping should be further studied on a wider scale in hemodialysis patients.

## Data Availability

The datasets generated and/or analysed during the current study are available from the corresponding author on reasonable request.

## Abbreviations

CD: Cluster of differentiation
ESKD: End Stage Kidney Disease
rRT-PCR: Real-Time Reverse Transcription-Polymerase Chain Reaction
SARS-CoV-2: Severe Acute Respiratory Syndrome coronaVirus 2
Treg: T regulator lymphocytes
